# *Bronchial Injections*: A Report, with a Statistical Table, of One Hundred and Six Cases of Pulmonary Diseases Treated by Bronchial Injections

**Published:** 1856-04

**Authors:** 


*Bronchial Injections:* A Report, with a Statistical Table, of one hundred and six cases of Pulmonary Diseases treated by bronchial injections. By HORACE GREEN, M. D., LL. D., etc., etc. From the American Medical Monthly.

This pamphlet is another evidence of that industry and energy with which Dr. Green pushes his favorite treatment of pulmonary disease. In it are reiterated the assertions of previous papers, and a new array of cases is brought up to fortify his doctrine. We insert, *in extenso*, his analysis of results, that our readers may have a fair idea of what is claimed to be performed:

"If we analyze the *one hundred and six* cases reported in the following table, it will be found that *seventy-two* of the sum total have been recorded as cases of *tuberculosis*. Of this number, *thirty-two* were considered cases of *advanced phthisis*—cases in which tubercular cavities were recognized in one or both lungs, and *thirty-nine* cases of *early phthisis*. Of the first division—advanced phthisis, *fourteen* have since died. *Twenty-five* were more or less improved, their lives being apparently prolonged by this means of medication. *Seven* only of the thirty-two cases of advanced phthisis were not benefitted by the injections.

"Of the *thirty-nine* cases of *incipient tuberculosis*, *twelve* of this division have apparently recovered. *Five* more of this number are now, or were at the last report, in the enjoyment of a good degree of health. These five cases were classed by Dr. Richards with the twelve recoveries, making *seventeen* in all. But, as there is more doubt respecting the cases of these five than of the first twelve, I have not retained them in the class of cases cured.

"With respect to the above twelve cases, I say *apparently* cured; for, although the appearance of these patients, as manifested both by the physical and rational signs, is indicative of an ordinary degree of health, yet, in a disease like that of tuberculosis, every medical man is aware that one year is a period too brief to speak decidedly with regard to the positive and final result.

"Of the remaining *twenty-two* cases, many of whom are still under treatment, *seventeen* have been greatly improved by topical medication; *three* more have been moderately benefitted; while *three* only have failed to obtain any advantage from the local measures which have been adopted.

"Of the *twenty-eight* cases of *bronchitis*, *sixteen* have been dismissed cured, or so much improved as to require no further treatment. All the others have been greatly benefitted, although some are still under treatment.

"Bronchial injections have been employed in six cases of asthma only. In the treatment of this disease, the application of a solution of the nitrate of silver, by means of the sponge-armed probang, to the larynx, will in most cases, it has been found, prove more certain and efficient in its effects than catheterism of the air-passages. Hence, in nearly all the cases of this disease which have come under my observation, they have been treated by direct applications of the caustic solution to the larynx and trachea. It was only when this disease was complicated with bronchial inflammation that the flexible tube was employed.

"The six cases of asthma recorded in the table were all complicated with bronchial or pulmonary disease. In all except one the disease was removed by the use of bronchial injections. The single case not fully restored, was that of a lady from Ohio, who left greatly benefitted, after three applications only of the injecting tube."

It is, of course, known to our readers that Dr. Green's special treatment consists in the injection of medicated liquids (almost always a solution of nitrate of silver) into the affected lung. He claims to be able, not only to carry a sponge through the larynx into the trachea, but to push a flexible tube at will into either bronchus, and inject from it into a tubercular cavity at will.

The report of the committee of the Academy of Medicine, of New York, showed, conclusively, that a tube might be thus introduced, but that it was, to use Dr. Green's language, "an operation difficult of performance, under the most favorable circumstances—cannot be accomplished, 'and it should never be attempted, until the parts implicated are thoroughly *educated* by the necessary preparatory operations. These operations consist in cauterizing, successively, the pharynx, the opening of the glottis, and the larynx for several days, (even for weeks, if necessary,) before the introduction of the injecting tube into the trachea and bronchi.' "

Further than this the report of that committee showed that (and any person who has ever manipulated the region knows the fact) it is not always easy to tell *where* the tube may be. In various instances mistakes as to its location were made, in which even Dr. Green, familiar as he is with the operation, participated. On one occasion a patient *vomited* the contents of the stomach through a tube which Dr. Green had just averred to be in the trachea.

The fact is, however, sufficiently established, that a tube may be carried into the trachea. Whether it may be at will directed into either bronchus, is a question yet unsettled. That Dr. Green *believes* it may, we do not doubt. The natural tendency of a tube thus introduced would be down the left bronchus. It would require very skillful handling to pass it into the right, as near as we can judge from some failures of our own upon a dead body.

We do not ourselves attach any great weight to the anatomical difficulties urged against Dr. Green's operation. It is enough to say of them, that they are serious, but not insuperable. It can never be an operation of common occurrence, for few will be adepts enough to get the tube through the larynx, and of those few, still fewer will ever acquire the skill to traverse either bronchus at will, or *to know which one they do occupy.* In fact we think this latter a very puzzling question to settle.

We are willing to grant, then, that, with occasional liability to mistake the tube may be introduced, by skillful operators, and that it may, *perhaps,* be known which bronchus it follows. We have left, then, a question for fair and quiet discussion, viz:

Are injections of solutions of nitrate of silver, or other topical remedies, likely to result in benefit to the patient?

We would be glad to concede the affirmative at once, but are unable to do so for several reasons.

1st. The table given by Dr. Green, as well as the thirteen cases reported at length, does not indicate the general remedial, hygienic, or dietetic treatment of the patients. The question at once arises: how much of the success gained was due to the injection, and how much the general treatment?

Inasmuch as Dr. Green's paper furnishes but very trivial data from which to judge, we are at liberty to form our own opinion. We take it for granted that a practitioner of Dr. Green's acumen would not treat a *cutaneous* disease, involving constitutional vice, with local remedies alone. He would, of course, call to his aid the weapons of internal medication, hygiene and diet. If he would be governed by this reasoning in a simple *cutaneous* disease, how much more would he need all possible aids in the treatment of tuberculosis, a disease not of the lungs but of assimilation! It would be accusing him of criminal recklessness to assume that the injection was the only, or the most important treatment. It is not possible that he rejects those kindly and grateful means, which in the hands of other practitioners result in a success equal to his own. On another page of this journal will be found a few remarks on the curability of consumption, written before Dr. G.'s pamphlet was received. May we not refer to it as some proof—we might have made it stronger—that there are general remedies too important, too successful, to be rejected by any prudent or honest practitioner? And if we are right in assuming that Dr. Green *does* make use of them, can we not also suppose that he may have given to the probang some portion of the credit due to the oil, the fat, the exercise and the ale?

So much for our first objection. We can only add, that while we regret that Dr. G. should have been so sparing of information on this important means of judging the merits of his treatment, we should still more regret to learn from him that he neglects those means which look, not only to the closing of a cavity, but to the prevention of further deposits of tubercle.

2d. Our second objection is, that we are, to a certain extent, unable to judge of the true character of many of his cases. In some of them this arises from a want of precision in the report of symptoms, and in others from an inaccurate pathology which leads us to question the value of the observations.

We take the case of Prof. Minor. The rational signs are such as to indicate confirmed phthisis; yet we find that he had "dullness," and not as one might suppose, either flatness or cavernous resonance, at the apex of the *right* lung. The chest expanded imperfectly on that side, we are told. "Expiration is also prolonged on this side, whilst the respiratory murmur is augmented in force under the left clavicle." Either of these conditions exists in about one-half of healthy chests.

"Bronchial räles are heard on both sides, while a severe cough, with large muco-purulent expectoration, which is occasionally streaked with blood, is present. Evidence of the presence of long-continued follicular disease exists, for the mucous crypts of the pharynx have disappeared, and the right tonsillary gland is entirely destroyed, and its place, between the anterior and posterior columns, is occupied by a large deep ulcer."

This case is reported as one of bronchitis complicated with tubercles. We hazard nothing to our reputation as a diagnostician, in saying that, judging from Dr. Green's account, which of course is not softened, we believe it to have been pure bronchitis. If, as the writer of this pamphlet would have us to suppose, this Prof. Minor was cured of consumption by injections alone, without other treatment, the consumption *should have been proved.* The hue of the sclerotic, the shape of the fingers, the presence or absence of the red line upon the gums, the probability of hereditary taint, and the microscopic character of the sputa should have been noticed.

So, too, in the case of John Moore. The diagnosis is assumed, not proven. But to abandon an analysis of special cases which would be tedious, we find of that of nine cases of tubercle in which the signs are given at some length, six of them rest upon slight dullness beneath the *right* clavicle as the only sign, except that one of them is said to present "marked signs of cavity," while the other three are recorded as presenting "slight dullness" in *both*

apices, but most, in each case, upon the right. In not one of these cases are we satisfied with the proof of the existence of tubercle.

So far our objections apply only to tubercular cases. How is it in bronchitis?

Local means may certainly assume a high degree of importance in bronchial inflammation, and we are not prepared to dispute the accuracy of the report in this particular. Whether from our own unskillfulness, or from the unfortunate character of our cases, we have often failed in curing our cases of follicular pharyngitis by the nitrate of silver, in whatever strength used. Perhaps, in more skillful hands, different results may obtain, but neither in diseases of the throat, the uterus, or the eye, have we met with that uniform success which seems to have blessed the use of this remedy in other hands.

